# *HOXA7*, *HOXA9*, and *HOXA10* are differentially expressed in clival and sacral chordomas

**DOI:** 10.1038/s41598-017-02174-5

**Published:** 2017-05-17

**Authors:** Daniela Jäger, Thomas F. E. Barth, Silke Brüderlein, Angelika Scheuerle, Beate Rinner, Adrian von Witzleben, André Lechel, Patrick Meyer, Regine Mayer-Steinacker, Alexandra von Baer, Markus Schultheiss, Christian R. Wirtz, Peter Möller, Kevin Mellert

**Affiliations:** 1 Institute of Pathology, University of Ulm, Ulm, Germany; 2 Division of Biomedical Research, Medical University of Graz, Graz, Austria; 3 Department of Internal Medicine I, University of Ulm, Ulm, Germany; 4 Department of Dermatology, University of Ulm, Ulm, Germany; 5 Department of Internal Medicine III, University of Ulm, Ulm, Germany; 6 Department of Trauma Surgery, University of Ulm, Ulm, Germany; 7 Department of Neurosurgery, University of Ulm, Ulm, Germany

## Abstract

Chordomas are rare tumours of the bone arising along the spine from clivus to sacrum. We compared three chordoma cell lines of the clivus region including the newly established clivus chordoma cell line, U-CH14, with nine chordoma cell lines originating from sacral primaries by morphology, on genomic and expression levels and with patient samples from our chordoma tissue bank. Clinically, chordomas of the clivus were generally smaller in size at presentation and patients with sacral chordomas had more metastases and more often recurrent disease. All chordoma cell lines had a typical physaliphorous morphology and expressed brachyury, S100-protein and cytokeratin. By expression analyses we detected differentially expressed genes in the clivus derived cell lines as compared to the sacral cell lines. Among these were *HOXA7*, *HOXA9*, and *HOXA10* known to be important for the development of the anterior-posterior body axis. These results were confirmed by qPCR. Immunohistologically, clivus chordomas had no or very low levels of HOXA10 protein while sacral chordomas showed a strong nuclear positivity in all samples analysed. This differential expression of *HOX* genes in chordomas of the clivus and sacrum suggests an oncofetal mechanism in gene regulation linked to the anatomic site.

## Introduction

Chordomas are rare, malignant bone tumours thought to arise from notochordal remnants along the spine. This tumour entity is generally considered as a low-grade malignant tumour, but metastases and recurrences occur^1, 2^. Chordoma therapy usually consists of surgical resection in combination with radiotherapy. There is a lack of efficient standard chemotherapy, which may be due to the generally slow tumour growth^3, 4^. Therefore, there is a focus on novel therapeutic strategies for the treatment of chordoma, such as targeting the PDGF receptor^5–7^, VEGF receptor^8^ or mTOR-kinase^9, 10^. Overexpression of epidermal growth factor receptors (EGFR) has been reported to be a frequent event in chordoma^11–14^ and inhibition of the EGF receptor 1 has a significant effect on cellular growth^15, 16^. As the majority of chordomas show a deletion of the tumour suppressor gene *CDKN2A* (encoding for the protein p16)^17, 18^, we provided evidence that the CDK4/6 inhibitor palbociclib may be a promising therapeutic option for chordoma treatment^19^. Up to now, none of these strategies have made it to clinical usage.

A characteristic feature of chordoma is the overexpression of the transcription factor T encoding for the protein brachyury. Brachyury is essential for the development of the notochord and the formation of posterior mesodermal elements^20–22^.

Regarding developmental processes, an important role in the formation of the anterior-posterior body axis and other morphogenetic processes is assumed for members of the *HOX* gene family, a highly conserved gene family which encodes for several transcription factors^23, 24^.

In vertebrates, 39 *HOX* genes have been identified which are organized in four clusters, called *HOXA*, *HOXB*, *HOXC*, and *HOXD*. Each cluster is located on a different chromosome, namely 7p14, 17q21, 12q13, and 2q31, respectively. With regard to the formation of the anterior-posterior body axis, *HOX* gene expression follows the rules of the so-called temporal and spatial collinearity. This means that genes located at the 3′ end of the cluster are expressed earlier in the embryogenesis and have a more anterior boundary of expression than those located at the 5′ end^25–27^. As the two major predilection sites of chordoma are the cranial (clivus) and the caudal (sacral) ends of the spine, a comparison of these different entities might give new insights into the formation of chordomas. Due to the rareness of this tumour, there are a small number of chordoma cell lines and especially just two from clivus chordoma. In the following, we addressed the question whether differences between clival and sacral chordomas exist. We established a novel chordoma cell line, U-CH14, derived from a clivus chordoma, analysed this cell line together with two other clivus derived cell lines, MUG-CC1 and UM-Chor1, and compared them with nine sacral chordoma cell lines on genomic, gene expression and protein grounds to identify possible differences with focus on *HOX* genes. Finally, we validated these findings in our chordoma tissue bank *in situ*.

## Results

### Chordomas of the clivus differ in clinical features from sacral chordomas

Analyses of our chordoma tissue cohort (n = 43) with regard to eventual differences between chordomas of the clivus (n = 5) and the sacrum region (n = 24) showed that clivus chordomas were significantly smaller than sacral chordomas (p = 0.04). The median tumour size was 1.4 cm (inter quartile range (IQR): 0.65 cm; 2 cm) versus 7.0 cm (IQR: 5.125 cm; 14.65 cm). Clivus chordoma patients tended to be younger than sacral chordoma patients (median age at onset of disease: 49 years (IQR: 40; 80) versus 70 years (IQR: 51; 75.75)). Regardless of the different localisation along the spine, chordomas were of the ‘not otherwise specified’ (NOS)-subtype. The proliferation rate was lower in clivus chordoma samples, as all cases had a Ki-67 index lower than 10%. In sacral chordomas, 5/24 samples had Ki-67 indices above 10%. In p53 immunohistochemistry, 0/5 clivus and 7/23 sacrum chordoma cases showed a nuclear positivity in more than 1% of total cells (Supplementary Table 1, Supplementary Figure 1A+B). In deep targeted sequencing, all analysed patient probes (n = 22) harboured the common *TP53* variant p.P72R (Supplementary Table 1). In addition, one clival and two sacral chordoma cases carried mutations, namely p.P72S (3/22) and p.A76V (1/22). In our cohort, metastases occurred in sacral (8/24; lung, liver and lymph node), but not in clivus chordoma patients (0/5). Recurrent disease was found in 13/24 sacral and in 0/5 clivus chordoma patients. Due to the anatomical complexity of the site of clival chordomas only an incomplete resection status (R2) was achieved in 4/5 cases; in one case the resection status was uncertain (Rx) (Table 1).

**Table 1:** Summarized clinical data of the clival and sacral chordoma patients.

Patient	Localisation	Age at diagnosis	Gender	Tumour Size (cm)	Histological subtype	Ki-67	Metastases	Recurrences	R-Status
C1	Clivus	35	male	0.4	NOS	≤10%	—	—	R2
C2	Clivus	45	female	2.3	NOS	≤10%	—	—	Rx
C3	Clivus	49	female	0.9	NOS	≤10%	—	—	R2
C4	Clivus	81	male	1.4	NOS	≤10%	—	—	R2
C5	Clivus	79	male	1.7	NOS	≤10%	—	—	R2
S1	Sacrum	60	female	12	NOS	≤10%	—	—	Rx
S2	Sacrum	78	female	18	NOS	≤10%	—	—	R1
S3	Sacrum	50	female	10	NOS	≤10%	—	—	R0
S4	Sacrum	74	male	7	NOS	≤10%	—	—	Rx
S5	Sacrum	45	female	7	NOS	≤10%	—	1	R1
S6	Sacrum	68	male	6	NOS	≤10%	—	1	R1
S7	Sacrum	69	female	17	NOS	≤10%	1	—	R1
S8	Sacrum	45	male	20	NOS	≥10%	1	1	R1
S9	Sacrum	70	female	13.6	NOS	≤10%	—	1	R0
S10	Sacrum	76	female	6	NOS	≤10%	1	1	R0
S11	Sacrum	74	female	13	NOS	≤10%	—	1	R1
S12	Sacrum	71	female	18	NOS	≤10%	—	1	R1
S13	Sacrum	70	male	3	NOS	≤10%	1	—	R2
S14	Sacrum	67	male	6	NOS	≥10%	1	>1	Rx
S15	Sacrum	75	male	6.5	NOS	≤10%	—	1	R1
S16	Sacrum	49	male	5.2	NOS	≥10%	>1	>1	NA
S17	Sacrum	77	male	2.5	NOS	≤10%	1	1	R2
S18	Sacrum	78	female	5.1	NOS	≤10%	—	1	R1
S19	Sacrum	54	male	4.5	NOS	≥10%	—	1	R2
S20	Sacrum	47	male	2	NOS	≤10%	—	—	R2
S21	Sacrum	29	male	2.9	NOS	≤10%	1	—	R1
S22	Sacrum	71	male	8	NOS	≤10%	—	—	R0
S23	Sacrum	83	male	50	NOS	≥10%	—	—	R2
S24	Sacrum	79	female	15	NOS	≤10%	—	—	R0

*NOS* not otherwise specified, *R*-*Status* residual tumour status, *R0* no residual tumour, *R1* tumour cells detected microscopically at the resection margin, *R2* macroscopic detectable residual tumour, *Rx* R-status uncertain.

### Characterization of the clivus chordoma cell line U-CH14

**Figure 1:**
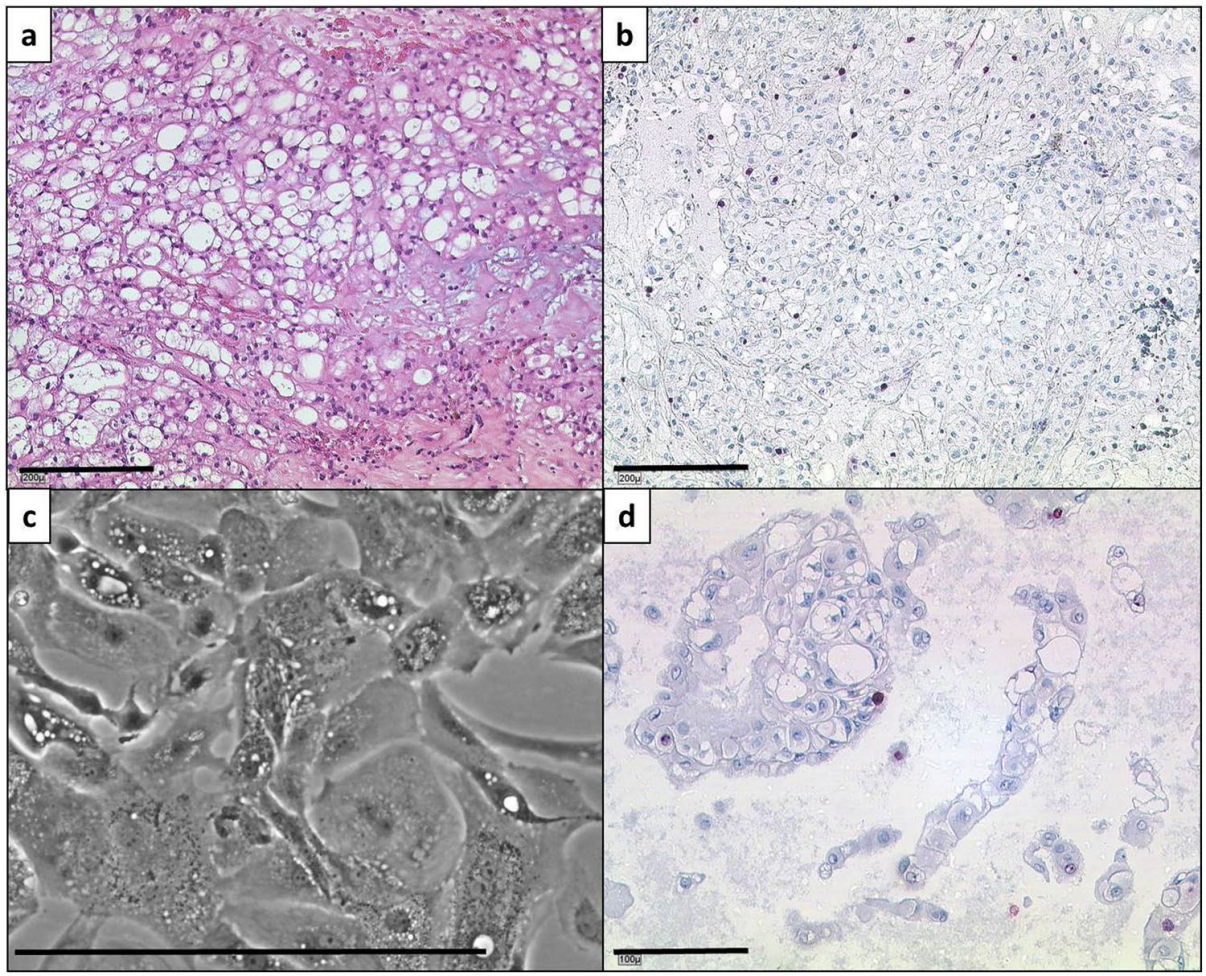
Morphology and proliferation rate detection of the parental tumour and the U-CH14 cell line. (**a**) Section of the parental tumour. The cells show the typical physaliphorous appearance. The tumour is classified as not otherwise specified (NOS) subtype. (**b**) Ki-67 staining of a parental tumour section. The proliferation index is below 5%. (**c**) Phase contrast image of the U-CH14 cell line. The typical morphology is conserved *in vitro*. (**d**) Proliferation index of U-CH14 (<5%; Ki-67 staining). Lines indicate 200 µm (**a**+**b**) or 100 µm (**c**+**d**).

After a cultivation period of about 2 years, U-CH14 was established as a stable chordoma cell line. Comparable to the primary tumour, the cells showed the typical physaliphorous appearance *in vitro* (Fig. 1c) and a generation time of about 10 days. The Ki-67 index representing the proliferation rate was below 5% both *in situ* and *in vitro* (Fig. 1b,d). Therefore, the long population doubling time of the cell line fitted to the slow tumour growth.

**Figure 2:**
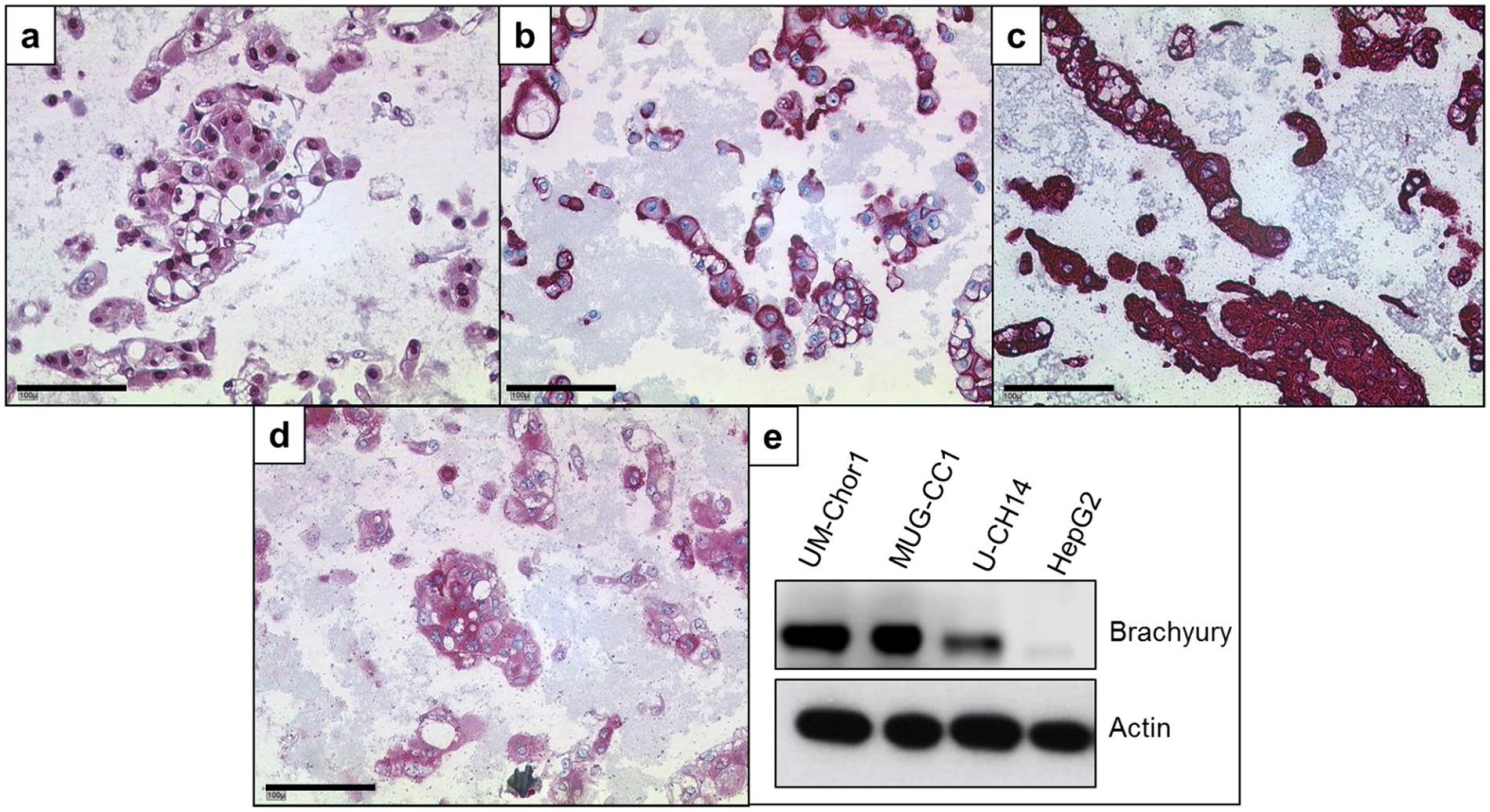
Immunocytochemistry and western blot analysis of U-CH14. The cells are strongly positive for brachyury (**a**), vimentin (**b**), cytokeratin (AE1/AE3) (**c**), and S-100-protein (**d**). (**e**) Western blot confirmation of the brachyury expression in the three clivus chordoma cell lines (UM-Chor1, MUG-CC1, and U-CH14). HepG2 served as a negative control. Lines indicate 100 µm (**a–d**).

Immunocytochemically, the tumour cells showed a strong nuclear expression of brachyury and a cytoplasmatic positivity of vimentin, cytokeratins, and S100-protein (Fig. 2a–d). Hence, all typical chordoma markers were conserved in the cell line. The expression of brachyury was verified by western blot analyses (Fig. 2e). The origin of the cell line was confirmed by STR comparison with its parenteral tumour. The same alleles could be confirmed in 10/12 markers. In U-CH14, the markers *D18S51* and *TH01* showed only one of the two alleles detected in the primary tumour indicating a loss of heterozygosity (Supplementary Table 2). Since *CDKN2A* gene loss is frequent in chordomas, the *CDKN2A* status was analysed by fluorescence *in situ* hybridization (FISH). A loss of *CDKN2A* in the chordoma cells was found as compared to the co-hybridized centromere 12 probe (Supplementary Table 3). Additional chromosomal gains and losses were determined by array comparative genomic hybridization (aCGH) analyses. Ten chromosomes showed alterations. We detected gains on chromosomes 2, 7, 9, 11, 14, 18, and 22 and losses on chromosomes 8, 9, 11, 17, 18, 20, and 22 (Supplementary Figure 2). The complete list of genes in the affected chromosomal regions is given in Supplementary Table 4. A total loss of chromosomal material was solely detected for chromosome 9p21.3-ter which confirms the total *CDKN2A* loss as identified by FISH analyses.

### Clivus chordoma cell lines differ in gene expression from sacral chordoma cell lines

**Figure 3:**
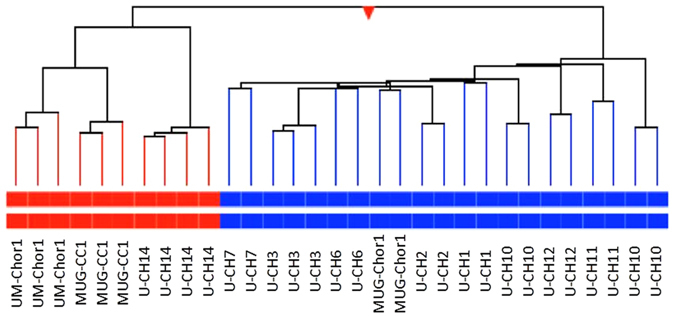
Hierarchical cluster analysis of nine sacral (blue) and three clivus (red) chordoma cell lines. The gene expression was tested using the 4 × 44 K whole genome microarray (Agilent Technologies). Each cell line was tested at least in duplicates.

Measurement of the expression of ~20000 transcripts showed differences between the clival and the sacral chordoma cell lines. Hierarchical cluster analysis of the three clivus (GEO accession number GSE95084) and the nine sacral chordoma cell lines (GEO accession number: GSE68497) revealed that the clivus chordoma cell lines clustered together in one group against the background of the sacral chordoma cell lines (Fig. 3). Filtering for most significant differentially expressed genes (p < 0.005), we identified 4572 genes being differently regulated in clivus chordoma cell lines. Pathway analysis (IPA) showed significant differences in the expression of more than 1000 genes related to physiological system development and function such as organismal development (282), tissue development (548), reproductive system development (85) and embryonic development (155). Gene ontology (GO) analyses revealed these differently expressed genes belonging to 25 distinct GO terms. Interestingly, in 12 of the 25 GO terms we found several members of the *HOX* gene family being down regulated in the clivus chordoma cell lines as compared to the sacral chordoma cell lines. Furthermore, in seven of the remaining GO terms at least one member of the *HOX* gene family was dysregulated. The common feature of all these GO terms was their impact in developmental processes, involving the anterior-posterior pattern formation and the skeletal development (Table 2).

**Table 2:** *HOX* genes down regulated in clivus chordoma cell lines compared to sacrum chordoma cell lines and the related gene ontology (GO) terms.

GO Term	Downregulated HOX genes in clivus cell lines
Anterior-posterior pattern formation	*HOXA2*, *HOXA4*, *HOXA5*, *HOXA7*, *HOXA9*, *HOXA10*
	*HOXB3*, *HOXB7*
	*HOXC9*
Skeletal development	*HOXA2*, *HOXA4*, *HOXA5*, *HOXA7*, *HOXA9*, *HOXA10*
	*HOXB3*, *HOXB7*
	*HOXC9*
Regionalization	*HOXA2*, *HOXA4*, *HOXA5*, *HOXA7*, *HOXA9*, *HOXA10*
	*HOXB3*, *HOXB7*
	*HOXC9*
Multicellular organismal development	*HOXA2*, *HOXA4*, *HOXA5*, *HOXA7*, *HOXA9*, *HOXA10*
	*HOXB3*, *HOXB7*
	*HOXC9*
Embryonic skeletal system morphogenesis	*HOXA2*, *HOXA4*, *HOXA5*, *HOXA7*
	*HOXB3*, *HOXB7*
	*HOXC9*
Developmental processes	*HOXA2*, *HOXA4*, *HOXA5*, *HOXA7*, *HOXA9*, *HOXA10*
	*HOXB3*, *HOXB7*
	*HOXC9*
Anatomical structure development	*HOXA2*, *HOXA4*, *HOXA5*, *HOXA7*, *HOXA9*, *HOXA10*
	*HOXB3*, *HOXB7*
	*HOXC9*
System development	*HOXA2*, *HOXA4*, *HOXA5*, *HOXA7*, *HOXA9*, *HOXA10*
	*HOXB3*, *HOXB7*
	*HOXC9*
Tissue development	*HOXA5*
	*HOXB3*

### Sacral chordomas have higher expression levels of *HOXA7*, *HOXA9*, and *HOXA10* mRNA than clivus chordomas

**Figure 4:**
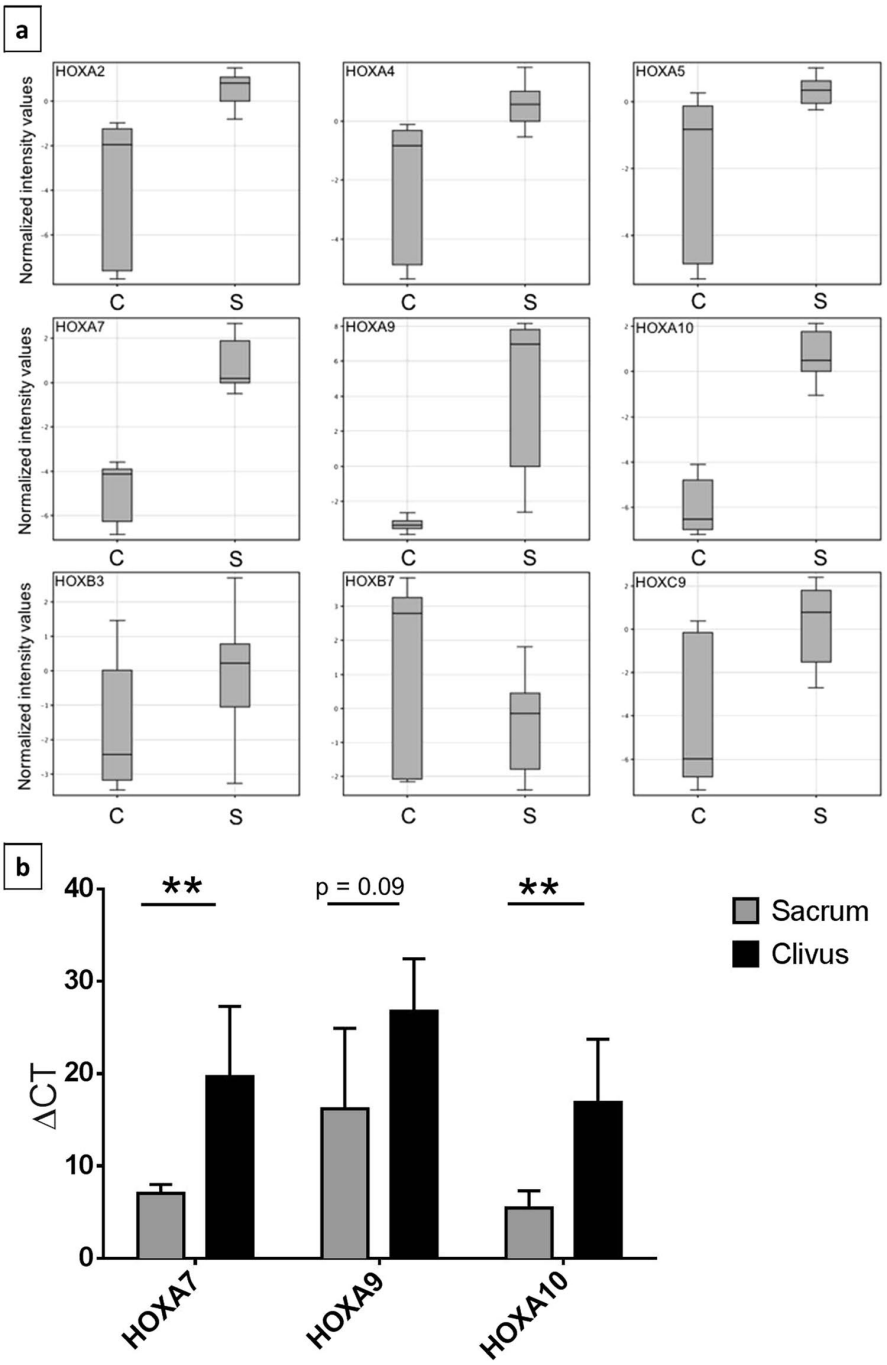
Expression of several *HOX* gene family members. (**a**) Box plot illustration of the differences in the expression of *HOXA2*, -*A4*, -*A5*, -*A7*, -*A9*, -*A10*, -*B3*, -*B7*, and -*C9* between clivus (**c**) and sacrum (S) chordoma cell lines. Measurements represent the results of microarray expression profiling of three clivus and nine sacrum chordoma cell lines (each cell line was tested at least in duplicates). (**b**) Validation of the expression of *HOXA7*, -*A9* and -*A10* in the chordoma cell lines *via* qPCR shown as measured ΔCT values. High ΔCT values represent lower expression of the corresponding gene. Measurements were performed in triplicates. Differences in expression were tested by t-tests. p < 0.05 was regarded as significant difference (*). **p < 0.01.

We analysed the expression of the different *HOX* genes in our two subgroups (Fig. 4a). As indicated by the corrected p-values, the most promising differentially expressed genes were *HOXA7*, *HOXA9* and *HOXA10* (p_corr_ 8.91 × 10^−10^; 3.31 × 10^−4^; 7.14 × 10^−12^). Compared to the sacrum lines *HOXA7* and *HOXA10* were downregulated in all clivus lines. For *HOXA9* expression we found a broad range of expression levels within the sacral subgroup. All three clivus cell lines, however, had very low expression levels. Therefore, these 3 *HOX* family members were further analysed in detail.

**Figure 5:**
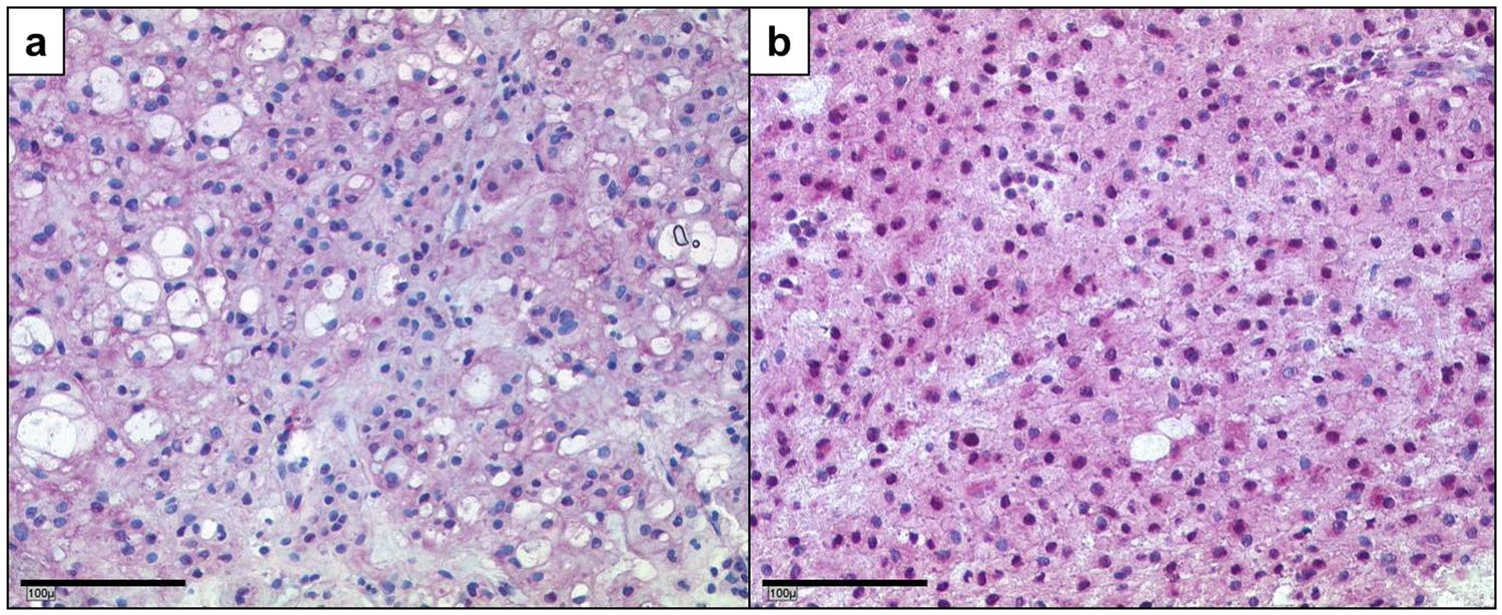
Differences of HOXA10 protein levels in clival and sacral chordomas. Immunohistochemistry of HOXA10 shows a very weak cytoplasmic staining of HOXA10 protein in clivus (**a**) and a nuclear positivity in the sacrum chordomas (**b**).

We verified the expression differences using qPCR (Fig. 4b). Whereas the low expression of *HOXA7* and *HOXA10* was an invariable feature of the clivus cell lines, a low expression of *HOXA9* was detected in all clivus lines and in 4/9 sacrum cell lines. The sacrum lines U-CH1, U-CH6, U-CH12, and MUG-Chor1 expressed *HOXA9* at low levels comparable to the clivus lines U-CH14, MUG-CC1, and UM-Chor1 (Supplementary Figure 3). We further analysed the *in situ* protein expression of HOXA10 in the 24 sacral and five clivus chordoma patients from our chordoma tissue bank by immunohistochemistry (as shown in Fig. 5). A strong nuclear positivity (+++) was detected in the majority of tumour cells in 20/24 sacral chordoma cases whereas the clivus cases had low levels of HOXA10 or were completely negative (Table 3).

**Table 3:** Expression of HOXA10 in correlation to the localisation of the tumour.

Patient Number	Localisation	Expression of HOXA10
C1	Clivus	−
C2	Clivus	−
C3	Clivus	+
C4	Clivus	+
C5	Clivus	+
S1	Sacrum	++
S2	Sacrum	++
S3	Sacrum	+++
S4	Sacrum	+++
S5	Sacrum	+++
S6	Sacrum	+++
S7	Sacrum	+++
S8	Sacrum	+++
S9	Sacrum	++
S10	Sacrum	++
S11	Sacrum	+++
S12	Sacrum	+++
S13	Sacrum	+++
S14	Sacrum	+++
S15	Sacrum	+++
S16	Sacrum	+++
S17	Sacrum	+++
S18	Sacrum	+++
S19	Sacrum	+++
S20	Sacrum	+++
S21	Sacrum	+++
S22	Sacrum	+++
S23	Sacrum	+++
S24	Sacrum	+++

Clivus chordomas (n = 5) have a weaker expression of HOXA10 than sacrum chordomas (n = 24) shown by immunohistochemistry.

## Discussion

Chordoma is a primary bone tumour with characteristic genomics, mRNA profiles, and expression of proteins such as brachyury, S100-protein, and the epithelial markers epithelial membrane antigen (EMA), and cytokeratins. Chordomas may arise all along the spine, ranging from the cranial to the caudal ends. Here, we present a comparison of chordomas of the clivus and the sacrum region. Analysing the clinical aspects of our chordoma tissue cohort, no significant differences could be detected with regard to morphology of the tumours and age of onset of disease. In contrast, the tumour entities differed clearly in size, proliferation rate, and the propensity to metastasize and recur. Obviously, clival chordomas are smaller than the sacral tumours explained by the localisation itself and earlier symptoms they provoke^28^. In contrast, sacral chordomas remain longer asymptomatic, serious limitations in the daily life, e.g. incontinency or vast ulcerations usually occur, but in late stages of the disease^29^. Otherwise, the small size of the clival tumours may be in parts explained by the lower proliferation rate detected in our cohort, suggesting a less aggressive behaviour. This hypothesis fits to our data concerning the absence of metastases and recurrent disease although in all cases the resection was rated as incomplete. This is in line with published data stating that sacrum chordoma metastasize two to fourfold more often as clivus chordoma^30–32^. The p53 staining of our cohort revealed that only sacral cases showed higher expression indices matching the finding that a strong nuclear labeling of p53 in IHC negatively correlates with progression free survival^33^. All examined cases showed the same *TP53* variant (p.P72R), as previously described by Fischer *et al*.^34^. Furthermore, we identified 2 additional mutations in 3 chordoma cases (resulting in p.P72S in C4, S23 and S16 and additionally p.A76V in the latter). These variants were graded neutral in 8/8 protein prediction tools (Supplementary Table 4). A correlation between the variants and enhanced nuclear p53 accumulation could not be found. Therefore, the differences in p53 protein expression in clival and sacral cases in our cohort are not due to the TP53 mutation status.

Our results argue for biological differences between clival and sacral chordomas as two different tumour entities. To further elucidate this, we established a novel cell line (U-CH14) derived from a clival chordoma in addition to the only two further clival chordoma cell lines available, MUG-CC1 and UM-Chor1. Morphology and the expression of typical chordoma markers are conserved in U-CH14 indicating that this cell line is a suitable *in vitro* model. Hence, we were able to compare three clivus with nine sacral chordoma cell lines on the expression profile level. In an unsupervised approach the clivus chordoma cell lines clustered together in one group and clearly separated from the sacral cell lines. This confirms that, indeed, there are differences between these two groups. Taking a closer look on the differently expressed genes it is remarkable that many of the downregulated genes in clival cell lines can be assigned to developmental processes. As chordoma cell lines have shown to express typical stem cell markers^35^, a connection to developmental processes seems to make sense. Amongst the downregulated genes in clivus chordomas, there were genes of the *HOX* family. *HOX* genes play a fundamental role in the development of the anterior posterior body axis^24^. The downregulation of *HOX* genes was validated in qPCR and confirmed in our tissue cohort.

Whether the differential *HOX* gene expression between clival and sacral chordoma is solely oncofetal in origin or has a pathologic role in the phenotype of the more aggressive sacral chordomas is an open question. Considering the redundancy within paralogue groups and the temporal and spatial collinearity, *HOX* expression effects solely caused by the localisation of the tumour would result in most of the 3′- *HOX* genes being up and 5′-*HOX* genes (including further members of the paralogue groups) being downregulated in clival chordoma and vice versa. The downregulated *HOX* genes in clivus cell lines (*HOXA2*, -*A4*, -*A5*, -*A7*, -*A9*, -*A10*, -*B3*, -*B7*, and -*C9*, Table 2) are distributed along the whole cluster and do not belong to corresponding paralogue groups. Therefore, it can be assumed that these *HOX* gene dysregulations cannot be explained by typical embryogenesis expression patterns and therefore not by the localisation of the originating tumour. Nonetheless, a final proof of this assumption can only be provided by systematically changing the expression of the identified *HOX* genes by overexpression or knockdown *in vitro*. Notably, most of the dysregulated *HOX* genes in clivus chordomas belong to the *HOXA* cluster located on chromosome 7p, a chromosomal region which frequently shows gains in chordoma cell lines^36, 37^. Hence, we analysed whether the clivus chordoma cell lines lack the typical gain of 7p, as explanation of a lower expression of *HOXA* cluster genes compared to sacral chordoma cell lines. Two of three clivus cell lines showed a gain of 7p fitting to published data on five of six sacral chordoma cell lines^19^. Therefore, the downregulation of the described *HOX* genes is not caused by a loss of chromosomal material.

An altered expression of *HOX* genes has been observed in various cancer types. As postulated in the so called “oncology recapitulates ontology” hypothesis, the development of malignancies is directly linked to differentiation and development of cells^38, 39^. Fitting to this, the reexpression of *HOX* genes in tumours is a sign for dedifferentiation and reprogramming towards stem cell features in cancer cells. For example, expression profiling of 39 *HOX* genes in head and neck squamous cell carcinoma (HNSCC) compared to normal tissue revealed that 18 of the 39 genes showed an increased expression^40^. High expressions of *HOXA7*, *HOXA9*, and *HOXA10* are frequently seen in acute myeloid leukaemia (AML) and the overexpression of HOXA9 is linked to a poor prognosis^41–43^. An increased proliferation in human hepatocellular carcinoma has been described for the overexpression of *HOXA7* ^44^. Here, we present the first study of *HOX* genes in chordomas of different localisations along the spine. As a consequence of the rareness of this tumour disease, the low number of cases (n = 29) and cell lines (n = 12) may be a limiting factor of this study. Nevertheless, the expression of various *HOX* genes in sacral chordoma fits well with the results observed in other tumour entities. Furthermore, it was reported that the expression pattern of *HOX* genes can differ between cancer subtypes of one tumour entity. Abe *et al*. showed that in both squamous cell carcinoma (SCC) and adenocarcinoma of the lung, *HOXA5* and *HOXA10* are expressed at high levels, while *HOXA1* and *HOXC6* were only overexpressed in SCC^45^. The difference in *HOX* gene expression between clival and sacral chordomas buttresses our hypothesis of two separated subentities of chordomas. Sacral chordomas have a higher expression of *HOX* genes indicating a lower differentiation status which may be linked to tumour aggressiveness presenting itself by higher proliferation, the occurrence of metastases and recurrent disease, as seen in our chordoma cohort. As *HOX* overexpression is a feature in various cancer types, there is a high effort in trying to take advantage of *HOX* transcription factors as a potential therapeutic target. It is known that PBX forms dimers with many HOX proteins and potentiates the DNA binding capacity and specificity^46^. The interaction between HOX and PBX proteins was reduced by using a cell-permeable 18-amino acid peptide called HXR9 which induced apoptosis in cancer cell lines^47–49^ and tumour growth reduction in melanoma xenograft models^50^. Hence, HXR9 may also be a potential agent for treating sacral chordomas.

In summary, we here describe the newly established clivus chordoma cell line U-CH14, to increase the number of the existing clivus chordoma cell lines to three. In addition, we compared clival and sacral chordomas with regard to clinical features of our chordoma cohort and gene expression profiles of the existing cell lines. We found the expression of various *HOX* genes as a discriminator between these two subtypes.

## Material and Methods

### Cell culture and establishment of a novel chordoma cell line

A new clival chordoma cell line, U-CH14, was established from a 79-year-old, male chordoma patient. The tumour measured 1.7 × 1.4 × 1.2 cm and was localised in the clivus region and was resected *via* a transnasal endoscopic approach at the Department of Neurosurgery, Günzburg, Germany. Only a partial resection was achieved and the patient received proton beam therapy hereafter. Histologically, the tumour presented as a clivus chordoma of the not otherwise specified (NOS) subtype according to the WHO. The diagnosis was confirmed as the tumour showed immunopositivity for brachyury, S100-protein, and epithelial membrane antigen (EMA). The proliferation rate determined by the anti-Ki-67 antibody was below 5%.

The tumour tissue was immediately processed for cell culture at the Institute of Pathology at the University Hospital Ulm. The material was mechanically minced into small pieces and partially digested with collagenase. Isolated cells were cultured in Iscove’s Modified Dulbecco’s Medium/RPMI 1640 (4:1; Lonza, Basel, Switzerland) with 10% fetal bovine serum (Biochrom AG, Berlin, Germany), 2 mM glutamine, and penicillin/streptomycin. Before reaching a state of complete confluence, the cells were detached using trypsin/EDTA (0.25%/0.02%, Lonza). Another continuous chordoma cell lines, MUG-CC1 (Clivus) was contributed by Beate Rinner^37^. UM-Chor1 (Clivus) and the sacral chordoma cell line MUG-Chor1^51^ were provided by the Chordoma Foundation (www.chordomafoundation.org), the other sacral cell lines U-CH1, U-CH2, U-CH3, U-CH6, U-CH7, U-CH10, U-CH11, and U-CH12 were established at the Institute of Pathology, University Hospital Ulm, as previously described^19, 36, 52^. All cell lines were cultured applying the same protocol as mentioned above. All the performed methods and experiments were in line with the guidelines of the ethics committee of the Federal General medical Council (Dtsch Arztebl 2003;100: A1632 [Heft 23]; Dtsch Arztebl 2003; 100: A 2251 [Heft 34–35]). The research was carried out in compliance with the Helsinki Declaration. All experimental protocols were conducted and approved in accordance with the local ethics committee of the University of Ulm (votum for usage of archived human material 03/2014). The patient gave his written informed consent for scientific usage of his tumour cells.

### Immunohistochemistry

Immunohistochemistry was performed on formalin-fixed and paraffin-embedded tissue *via* the avidin-biotin-complex-method using the K005 AP/RED Detection System (Dako, Glostrup, Denmark). The following antibodies were used: monoclonal antibodies against brachyury (H-210, Santa Cruz, Dallas, USA, 1:100), epithelial membrane antigen (EMA; E29, Dako, 1:500), vimentin (VIM3B4, Dako, 1:300), cytokeratin (AE1 + AE3, Dako, 1:100), Ki-67 (MIB-1, Dako, 1:200), p53 (DO-7, Dako, 1:500) and the polyclonal antibodies against S100-protein (Dako, 1:1000) and HOXA10 (OriGene Technologies, Rockville, USA, 1:100). Appropriate positive and negative controls were included.

The ratio of positive chordoma cells was characterized as follows: “no immunoreactivity detected” (−), “immunoreactivity in up to 30% (+), “immunoreactivity in more than 30% and up to 70%” (++) and “immunoreactivity in more than 70%” (+++) of the total number of chordoma cells in the section^53^.

### Short tandem repeats (STR) analysis

For genotyping of the cell lines the GenomeLab STR Primer Set Kit (Beckman Coulter, Krefeld, Germany) and the Amplitaq GOLD DNA Polymerase (Life Technologies, Carlsbad, USA) were used, respectively. The analysis was performed according to the manufacturer’s handbook. PCR products were separated on the CEQ GeXP capillary electrophoresis system (Beckman Coulter). 12 alleles were determined using the Gene marker software (Softgenetics, State College, USA).

### Chordoma tissue bank

We analysed five clival and 24 sacral chordoma samples of our chordoma tissue bank (n = 43)^54^ with regard to eventual differences between clival and sacral chordomas concerning the age of the patients, tumour size, presence or absence of metastases and/or recurrences, R-status, proliferation rate, *TP53* status, and histological subtype.

### Gene Expression Analysis

Total RNA was extracted from the three clival chordoma cell lines (U-CH14, UM-Chor1, and MUG-CC1) and from the 9 sacral chordoma cell lines (U-CH1, U-CH2, U-CH3, U-CH6, U-CH7, U-CH10, U-CH11, U-CH12, and MUG-Chor1) using the RNeasy Mini-Kit (Qiagen, Hilden, Germany). Quality and quantity control was performed using the 2100 Bioanalyzer (Agilent Technologies, Santa Clara, USA). Gene expression analysis was performed using the Human Gene Expression 4 × 44 K Microarray Kit (Design ID 014850, Agilent Technologies). 200 ng of each sample was used and processed according to the standard protocol (Agilent Technologies). Each sample was analysed in duplicates at least. The signals were captured by a Microarray Scanner (G2565, Agilent Technologies, Santa Clara, USA). The generated data was analysed using the software Genespring 12.1 (Agilent Technologies) and Ingenuity Pathway Analysis (IPA; Qiagen). Hierarchical clustering was performed with an euclidean similarity measure and centroid linkage rule. Differentially expressed genes with statistical significance (p-value cut-off ≤ 0,005) between the two groups (clivus versus sacrum) were identified using scatter plot filtering. Classification of the differently expressed genes was performed via gene ontology (GO) analysis.

### Real-time polymerase chain reaction (qPCR)

Verification of the microarray results was performed using qPCR. Single-strand cDNA was synthesized from total RNA using the QuantiNova Reverse-Transcription Kit (Qiagen). qPCR was performed using the QuantiNova SYBR Green PCR Kit (Qiagen) and the Light-cycler Rotor Gene Q (Qiagen). The expression of the following genes was analysed: *HOXA7*, *HOXA9*, and *HOXA10* using the suitable QuantiTect Primer Assays (Qiagen). β-Actin and GAPDH served as an internal control. The PCR reaction was performed as follows: the initial denaturation step was 95 °C for 5 min, followed by 60 cycles at 95 °C for 10 sec and at 60 °C for 30 sec. All experiments were performed as triplicates.

### Array comparative genomic hybridization (aCGH)

Array comparative genomic hybridization was carried out using the SurePrint G3 Human CGH Microarray 8 × 60 K (Design ID 021924, Agilent Technologies). DNA isolation of the cell lines was performed using the QiAmp DNA Mini Kit (Qiagen). Quantity and quality of the DNA were assessed on the NanoDrop platform (Thermo Fisher Scientific, Rockford, USA). DNA hybridization was performed according to the standard procedures after labelling of 500 ng of the sample DNA and the control DNA (Human Reference DNA Female or Human Reference DNA Male, Agilent Technologies). Microarray data was analysed using Agilent Genomic Workbench (Agilent Technologies).

### Protein isolation and Western blot

Protein isolation was performed using TNT-buffer (20 mM Tris (pH 8,0), 200 mM sodium chloride, 1%-Triton-X100, 1 mM DTT) containing both phosphatase and protease inhibitor cocktails (Sigma-Aldrich, St. Louis, USA). Lysates were incubated in liquid nitrogen for 3 min and then centrifuged at 14 000 rpm at 4 °C to remove debris. The isolated proteins were quantified using the Biorad protein assay (Biorad, Munich, Germany) applying the Bradford method. SDS-PAGE was performed using NuPAGE 4–12% Bis-Tris protein gels (ThermoFisher Scientific). 10 µg of the protein lysate was used; after gel electrophoresis the proteins were transferred to a nitrocellulose membrane applying a standard semi-dry electro-transfer method. The membrane was blocked with TBS, 0.1% Tween 20, 10% skim milk powder (Sigma-Aldrich) for 60 min. Following, the membrane was washed thrice (each 10 min) with TBS containing 0,1% Tween 20 and then incubated with the primary antibody (Brachyury, H-210, Santa Cruz, 1:1000) overnight at 4 °C. After three washing steps the membrane was incubated for 1 hr with the appropriate secondary horseradish-peroxidase-conjugated antibody. After further washing, ECL detection was performed using the SuperSignal West Dura Extended Duration Substrate (ThermoFisher Scientific). Anti-β-actin antibody (AC-74, Sigma-Aldrich, St. Lewis, USA, 1:10000) was used to detect β-actin as a house keeping protein.

### FISH-Analysis

FISH was performed using the commercially available ZytoLight SPEC CDKN2A/CEN9 Dual Colour Probe (Zytovision, Bremerhaven, Germany) according to the manufacturer’s protocol.

### Next generation sequencing

For isolation of genomic DNA from the FFPE tissue samples, 5 µm tissue slices were transferred to glass slides. To spot the area containing the tumour, HE stained FFPE tissue slices (2 µm) were marked by an expert pathologist. The tumour-harboring areas of the FFPE tissue were subjected to a DNA extraction procedure using the GeneRead DNA FFPE kit (Qiagen) according to manufacturer’s instruction. DNA isolation of the cell lines was performed using the QiAmp DNA Mini Kit (Qiagen). DNA purity and concentration was determined fluorometrically (Qubit 2.0; Invitrogen). For molecular characterization of both tumour tissue and cell line DNA, we employed a targeted re-sequencing methodology using the GeneRead V2 chemistry (Qiagen) and a custom-made re-sequencing panel including primers for all exons of *TP53*. Target enrichment, amplicon processing, and library generation were performed according to the manufacturer’s handbook. For target enrichment, we included 1 ng to 40 ng DNA. Successful target enrichment and library generation was checked using the High Sensitivity DNA kit on a bioanalyzer device (Agilent Technologies). Libraries were diluted to 10 pM solutions and the sequencing was performed on a MiSeq platform (Illumina, San Diego, CA, USA) using the V2 chemistry. Mean read depth on target region was 200–4000 fold and 99% of bases were covered at 96–100% on average. The resulting fastq files were subjected to further analysis using the GeneRead web based analysis tool (http://ngsdataanalysis.sabiosciences.com/NGS2/), the Biomedical Workbench software package (Qiagen), and the Variant Studio software (Illumina). All identified mutations were manually re-analyzed using the Integrated Genome Viewer Software (Broad Institute, MA, USA). The impact of confirmed mutations on protein functions were classified using 8 online protein prediction tools (Polyphen-2, FATHMM, SIFT, PANTHER, Provean, PHD-SNP, SNAP, and Meta-SNP) according to the providers’ instructions.

### Statistics

For statistical analysis, student’s t-tests were performed. A p-value ≤ 0.05 was considered as significant.

## Electronic supplementary material

array CGH result table U-CH14

Supplementary Information
