# Supplementary material for: HOXA7, HOXA9, and HOXA10 are differentially expressed in clival and sacral chordomas
Source: Sci Rep. 2017 May 17;7:2032. doi: 10.1038/s41598-017-02174-5 (PMC5435709; doi:10.1038/s41598-017-02174-5)
Supplement: Supplementary file 2 — Supplementary Information [file 41598_2017_2174_MOESM2_ESM.pdf]

HOXA7, *HOXA9*, and *HOXA10* are differentially expressed in clival and sacral chordomas

Daniela Jäger*[1], Thomas FE Barth*[1], Silke Brüderlein[1], Angelika Scheuerle[1], Beate Rinner[2], Adrian von Witzleben[1], André Lechel[3], Patrick Meyer[4], Regine Mayer-Steinacker[5], Alexandra von Baer[6], Markus Schultheiss[6], Christian R Wirtz[7], Peter Möller[1], and Kevin Mellert[1]

1. Institute of Pathology, University of Ulm, Ulm, Germany
2. Division of Biomedical Research, Medical University of Graz, Graz, Austria
3. Department of Internal Medicine I, University of Ulm, Ulm, Germany
4. Department of Dermatology, University of Ulm, Ulm, Germany
5. Department of Internal Medicine III, University of Ulm, Ulm, Germany
6. Department of Trauma Surgery, University of Ulm, Ulm, Germany
7. Department of Neurosurgery, University of Ulm, Ulm, Germany

*these authors contributed equally

Supplementary Information

Supplementary table 1. *TP53* status in the chordoma tissue cohort and cell lines. Listed are labels of patients and cell lines, localisation, amount of positive cells in p53 immunohistochemistry, and the *TP53* mutation status. The distinct mutations resulting in amino acid changes are given. * indicates cases with low read depths. n.a. = not analysed.

Patient/Cell line	Localisation	p53 IHC (% positive)	*TP53* mutation status
C1	Clivus	<0.1	p.P72R
C2	Clivus	<0.1	p.P72R
C3	Clivus	<0.1	p.P72R
C4	Clivus	<0.1	p.P72R, p.P72S
C5	Clivus	<0.1	p.P72R
S1	Sacrum	<0.1	n.a.
S2	Sacrum	<0.1	n.a.
S3	Sacrum	0.1-1	n.a.
S4	Sacrum	<0.1	n.a.
S5	Sacrum	<0.1	n.a.
S6	Sacrum	<0.1	n.a.
S7	Sacrum	0.1-1	p.P72R
S8	Sacrum	1-5	p.P72R
S9	Sacrum	<0.1	p.P72R
S10	Sacrum	<0.1	p.P72R
S11	Sacrum	0.1-1	p.P72R
S12	Sacrum	1-5	p.P72R
S13	Sacrum	5-10	p.P72R
S14	Sacrum	5-10	p.P72R

S15	Sacrum	0.1-1	p.P72R*
S16	Sacrum	>10	p.P72R, p.P72S
S17	Sacrum	1-5	p.P72R
S18	Sacrum	1-5	p.P72R
S19	Sacrum	0.1-1	p.P72R
S20	Sacrum	<0.1	p.P72R
S21	Sacrum	<0.1	p.P72R, p.P72S, p.A76V
S22	Sacrum	<0.1	n.a.
S23	Sacrum	<0.1	p.P72R
S24	Sacrum	n.a.	p.P72R
U-CH14	Clivus	0.1-1	p.P72R
UM-Chor1	Clivus	>10	p.P72R
MUG-CC1	Clivus	0.1-1	p.P72R
U-CH1	Sacrum	>10	p.P72R*
U-CH2	Sacrum	1-5	None
U-CH3	Sacrum	>10	p.P72R
U-CH6	Sacrum	>10	p.P72R
U-CH7	Sacrum	1-5	n.a.
U-CH10	Sacrum	0.1-1	p.P72R
U-CH11	Sacrum	5-10	p.P72R
U-CH12	Sacrum	0.1-1	p.P72R
MUG-Chor1	Sacrum	0.1-1	p.P72R

Supplementary table 2. Short tandem repeat analysis of the cell line U-CH14 and the corresponding primary tumour. The detected alleles for 12 different markers confirmed the U-CH14 cell line to origin from the primary tumour of the patient.

	**U-CH14 cell line**		**Primary tumour**	
**Marker**	**Allele 1**	**Allele 2**	**Allele 1**	**Allele 2**
AMEL	X	Y	X	Y
D3S1358	17	17	17	17
D8S1179	13	14	13	14
TPOX	8	8	8	8
CSF1PO	10	11	10	11
Penta D	14	15	14	15
D13S317	10	12	10	12
D7S820	8	10	8	10
D16S539	11	12	11	12
Penta E	7	19	7	19
TH01	10	10	9	10
D18S51	14	14	14	18

Supplementary table 3. FISH analysis for *CDKN2A* of the cell line U-CH14. All examined cells (n=50) showed no copy of *CDKN2A* in combination with 1 (36 %), 2 (54 %) or 3 (10 %) signals referring to the centromere of the chromosome 9.

***CDKN2A* copies**	**Centromere copies**	**Percentage of cells (n=50)**
0	1	36
0	2	54
0	3	10

Supplementary table 4. Prediction of functional effects of the p53 variants P72R, P72S, and A76V. The results of the corresponding prediction tools are given. A green background indicates results interpreted as a neutral, red colour indicates a result interpreted as a damaging amino acid change.

**p53 variant**	**Polyphen-2**	**FATHMM**	**SIFT**	**Panther**	**Provean**	**PHD-SNP**	**SNAP**	**Meta-SNP**
P72R	0.107	-0.54	0.58	prob. benign	-0.23	0.13	0.64	0.13
P72S	0.001	0.26	0.98	prob. benign	0.929	0.083	0.335	0.086
A76V	0.002	-1.28	0.33	prob. benign	-0.216	0.089	0.48	0.097

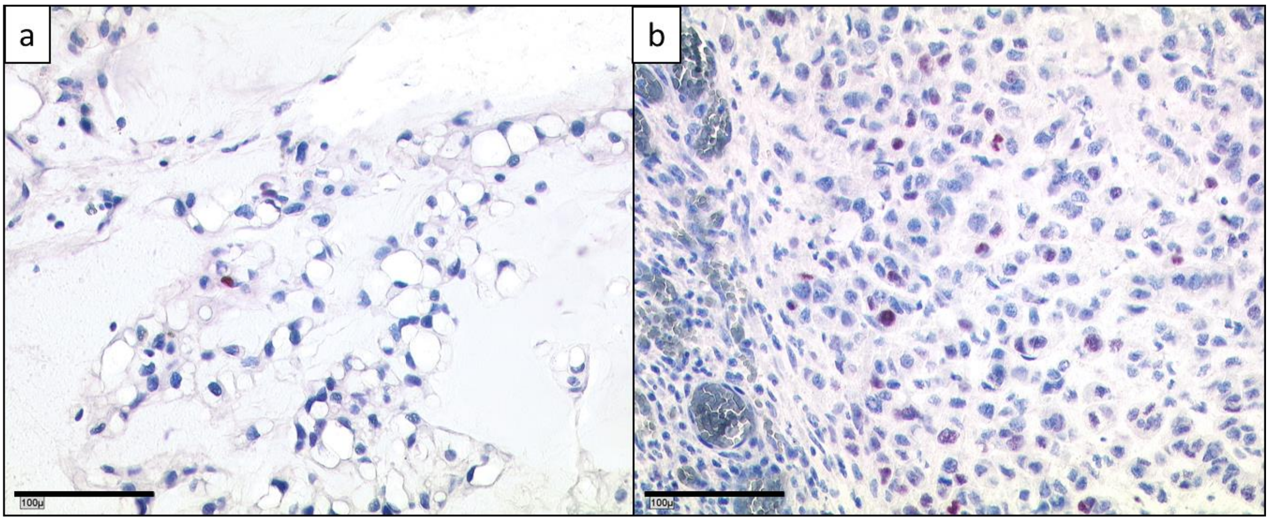

Supplementary figure 1. Immunohistochemical analysis of the expression of p53 in clival and sacral chordomas and cell lines. (a) Clival chordoma (C5) *in situ*. (b) Sacral chordoma (S16) *in situ*. Black lines indicate 100 µm.

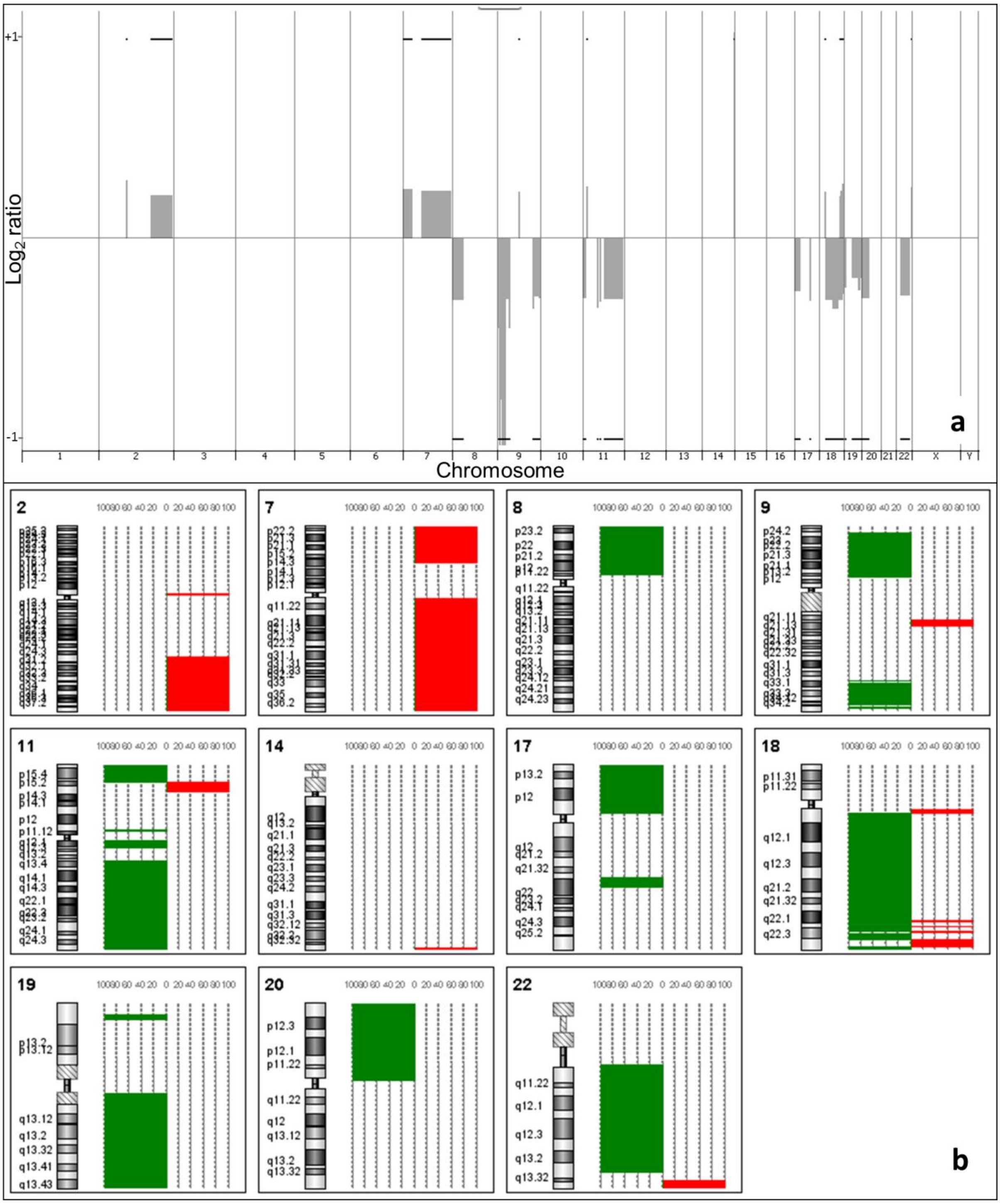

Supplementary figure 2. Array CGH karyogram of U-CH14. (**A**) Overview of gains and losses in the whole genome of U-CH14. The central line represents 2 copies of the highlighted region. Gains are illustrated upwards, losses downwards. The chromosomes are visualized from 1p (left) to Yq (right). (**B**) Ideogram summarizes genomic imbalances (gains: red; losses: green) of depicted chromosomes.

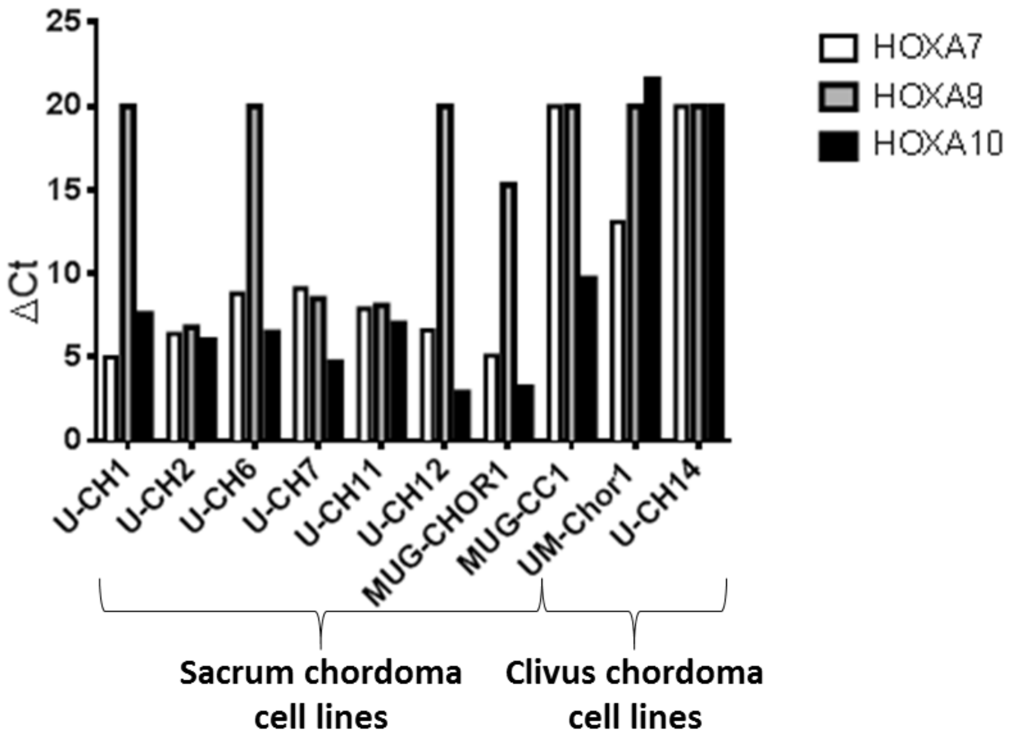

Supplementary figure 3. Expression analysis of *HOXA7*, *-A9* and *–A10* by qPCR in different chordoma cell lines. Shown are the relative expressions (indicated by delta cycle threshold (ΔCT) values).
